# Socially responsible investing through the equity funds in the global ownership network

**DOI:** 10.1371/journal.pone.0256160

**Published:** 2021-08-12

**Authors:** Takayuki Mizuno, Shohei Doi, Takahiro Tsuchiya, Shuhei Kurizaki

**Affiliations:** 1 National Institute of Informatics, Tokyo, Japan; 2 Hokkaido University, Sapporo, Japan; 3 Kyoto University of Advanced Science, Kyoto, Japan; 4 Waseda University, Tokyo, Japan; University of Burgundy, FRANCE

## Abstract

We analyze the connectivity of equity investments to the firms in the global ownership network that are reported as non-compliant with Environment, Social, and Government (ESG) benchmarks. We find that a large number of shareholders have ownership linkages to non-ESG firms, most commonly with three or four degrees of separation. Analyzing the betweenness centrality for shareholders connecting the ultimate owners and non-ESG firms, we find that the investment management companies play important roles in channeling the investment money into non-ESG firms, where largest American asset managers commonly have one to two degrees of separation on their ownership linkages to those problematic firms. Since asset managers collect capital from investors by running the equity funds, we analyze the ownership stakes and the associated voting rights attributable to the equity funds investors. We estimate the distribution of the power of corporate control over non-ESG firms among specific asset managers (such as BlackRock and Fidelity) and among different types of the equity funds (such as mutual funds and exchanged-traded funds), and explores how investing in the equity funds rather than ownership investing may have shifted the distribution of the power to control those non-ESG firms.

## 1 Introduction

We examine how equity stakeholders are linked to the companies considered non-compliant with the sustainable-investing standard. The practice of sustainable investing has recently gained its momentum. BlackRock, the largest asset manager in the world, has vowed to optimize its investing strategy with the environmental, social, and governance (ESG) benchmarks [1]. The International Campaign to Abolish Nuclear Weapons (ICAN), the 2017 Nobel Peace Prize laureate, calls for divestment from the production of nuclear weapons, arguing that between 2017 and 2019, 748 billion US dollars were invested in the nuclear weapons production by banks, investment companies, and pension funds [2]. However, is divestment sufficient for their goals? Research has shown that shareholders’ equity stakes extend their direct ownership shares and hence their corporate control may extend throughout the global ownership network [3, 4]. Moreover, research has emphasized the importance of the roles played by banks and other financial institutions in the corporate ownership network [3, 5, 6]. It is left unanswered, however, where the money the financial institutions use for their investments.

This study investigates the equity funds the investment firms use as their instruments to collect money and inject it to the corporate ownership network. To do so, we match up the data on the global ownership network to the data on equity funds as well as data on the ESG benchmarks. The combined dataset allows us to demonstrate that a number of shareholders (i.e., the ultimate owners) are indirectly linked in the global ownership network to the companies that are either responsible for producing military arms or negatively reported in the media or governmental sources with respect to environmental issues. Since our analysis reveals that large asset managers have betweenness centrality between the shareholders and the non-ESG firms, suggesting that they bridge equity investments and the non-ESG firms in the network, we then characterize the equity funds’ connectivity to, and control over, those problematic firms. Since asset managers and other institutional shareholders collect money through the financial instruments, they can own *more* shares of the target companies than otherwise. The size of ownership and control of the target companies is “inflated” to the degree that their purchase (and the ownership) of the share of target companies relies upon the collected money through the financial instruments.

To our knowledge, this study is the first of its kind to link the equity funds data to the shareholding network data and to describe the flow of equity stakes to non-ESG firms.

The remainder of this paper proceeds as follows. The next section describes the databases we use for our analysis of the ownership network, the equity funds, and the definition of what we call the “non-ESG firms” in this study. We also introduce the model we use to describe corporate controls and method of linking up the databases for the global ownership network and for the equity funds. Section 3 demonstrates the connectivity between the shareholders and the non-ESG firms in the global ownership network. Section describes not only the connectivity of equity funds to the non-ESG firms but also how the equity fund managers’ corporate power might be inflated by the influx of equity investments. Section 5 concludes this article with discussions on the directions of future research.

## 2 Data and method

### 2.1 Data on the global ownership network

Our data set on the global network of corporate ownership around the world consists of the shareholdings of 49 million corporations among 69 million shareholders (including firms and individuals alike) in 2016 obtained from *Bureau van Dijk’s Orbis* database [7]. Since the amount of sales of companies in this dataset aggregated at the country level are highly correlated with the distribution of national GDPs, we believe that the coverage of this dataset across countries is not biased if not completely exhaustive.

### 2.2 Data on equity funds

For the data on the equity funds, we utilize Refinitive’s *Lipper for Investment Management* database. Refinitive is a subsidiary of Thomson Reuters. The Lipper database covers 335,000 share classes including mutual funds, closed-end funds, exchange-traded funds (ETFs), hedge funds, pension funds, and insurance funds. For each fund, we use the information on the countries notified for sale, the fund manager, the stocks and bonds included in the portfolio (where we only use stocks), and the ownership information including the buyers and sellers. We calculate the proportion of shares included in each fund based on the market capitalization of each outstanding shares of the issuer.

### 2.3 Identifying non-ESG firms

Of the issues under the rubrics of Environment, Society, and Governance (ESG), our study focuses on the issues related to environment and military. For the ownership network and the equity funds, we use the data for December 2016, while for the companies that reportedly failed to adhere to the ESG standard we use the data for May 2020. This is because back in 2016 the notion of ESG investing was still in its nascent and hence the information on ESG investing was limited and the indicators had not been well established at that time. Thus, it is essential that we interpret the results in this article as the linkage and control possessed by shareholders and other equity stakeholders to the companies that later were identified as non-ESG compliant.

#### 2.3.1 Identifying non-green firms

We use Dow Jones Risk and Compliance’s Adverse Media Entities to flag the companies with environmental issues in the ownership network. This database lists companies that had adverse, negative media coverage on regulatory, social, and other risk-related topics, which uses governmental sources as well as information contained in Dow Jones’ Factiva articles. We use this list to flag the companies that had negative media coverage related to environmental issues as of May 2020.

#### 2.3.2 Identifying arms manufacturers

We use the sanction lists maintained by two governmental sources to identify the companies in the ownership network that are reportedly responsible for producing arms weapons. The first source is the “End User List” issued by Japan’s Ministry of Economy, Trade and Industry (METI) based on the Foreign Exchange and Foreign Trade Act (FEFTA) under international export control regimes. This list (which we refer to as the METI list hereafter) provides information on 546 entities from 14 countries and regions, for which concern cannot be eliminated regarding involvement in activities such as the development of weapons of mass destruction (WMDs) and other items [8]. The second is the so-called Entity List, which is published by the United States Department of Commerce’s Bureau of Industry and Security (BIS) as Supplement No. 4 to Part 744 of the Export Administration Regulations (EAR) [9]. This list, which we shall call the “BIS list,” provides information on entities who have engaged in activities that could result in an increased risk of the diversion of exported, reexported and transferred items to weapons of mass destruction (WMD) programs as well as activities sanctioned by the State Department as they are deemed contrary to U.S. national security and/or foreign policy interests. These sanction lists are also included Dow Jones Risk and Compliance’s Watchlist.

### 2.4 Linking the datasets

We use the following criteria to match up the shareholders and companies in the Orbis database (called *s*ource firms) with the companies that are identified on the METI/BIS lists (called *t*arget firms). We determine that the “source” firm *s* and the “target” firm *t* are identical if all the following four conditions are met. First, firms *s* and *t* are registered in the same country:

$$\begin{array}{c} c(s)=c(t). \end{array}$$ (1)

That is, the headquarters of firms *s* and *t* are located in the same country.

Second, the names registered for the source firm *s* and the target firm *t* are both sufficiently unique in terms of inverse-document frequency (idf) values:

$$\begin{array}{c} \text{IDF}(c,s)\geq \text{max}\{\text{idf}(c)\}\;\&\;\text{IDF}(c,t)\geq \text{max}\{\text{idf}(c)\}, \end{array}$$ (2)

where

$$\begin{array}{c} \text{IDF}\left(c,i\right)=\sum_{w\in W(i)}\text{idf}\left(c,w\right) \end{array}$$ (3)

gives the IDF value for firm *i* = {*s*, *t*} in country *c*, and

$$\begin{array}{c} \text{max}\{\text{idf}(c)\} \end{array}$$ (4)

returns the most unique keyword(s) (i.e., the keyword(s) with the highest idf value(s)) in the company names in country *c*. Note that

$$\begin{array}{c} \text{idf}\left(c,w\right)=\text{log}\frac{N(c)}{n(c,w)} \end{array}$$ (5)

on the right-hand side of (3) evaluates the importance (or uniqueness) of a keyword constituting its company name for each country *c*, where *N*(*c*) denotes the total number of firms in country *c* and *n*(*c*, *w*) denotes the number of the firms in *c* that include the term *w* in their company names.

Third, the keywords *w* with the highest idf value extracted each from the name registered for firm *s* (denoted by *w* ∈ *W*(*s*)) and the name registered for firm *t* (denoted by *w* ∈ *W*(*t*)) are identical:

$$\begin{array}{c} w(w\in W(s),\text{idf}(c,w)=\text{max}\{\text{idf}(c,s)\})=w(w\in W(t),\text{idf}(c,w)=\text{max}\{\text{idf}(c,t)\}); \end{array}$$ (6)

Fourth, the cosine similarity between vectors (of other keywords than the identical terms identified in the third step) **s** and **t** weighted by the idf values for firms *s* and *t* is sufficiently large: cos(*s*, *t*) ≥ 0.8, where

$$\begin{array}{c} \text{cos}\left(s,t\right)=\frac{s\cdot t}{\left\|s\|\cdot \|t\right\|}. \end{array}$$ (7)

This matching procedure produces 46,539 firms that have been reported to have experienced environmental problems. Similarly, 1,741 firms are identified as the problematic arms manufacturers. We have also checked the robustness of this matching procedure by changing the cutoff value used for the cosine similarity and by replacing the location of the headquarters of the firms with *city* or *postal code* if necessary. The results remain largely unchanged.

For matching the ownership data to the data on investment funds, the procedure is trivial. Since the Bureau van Dijk’s *Orbis* database and Refinitiv’s *Lipper* database provide the common ticker, we use this ticker to identify listed companies whose equity shares are included in each fund.

### 2.5 Ownership network, model of control, and algorithm

#### 2.5.1 Ownership network

Consider a *network* (*N*, *x*_*ij*_) consisting of a set of companies and their shareholders *N* = {1, 2, …, *n*}, where *i* ∈ *N* and *j* ∈ *N* respectively index a shareholder (be it individuals, corporations, or governments) and a company whose share is owned, directly or indirectly, by *i*. An *ownership linkage* *x*_*ij*_ ∈ [0, 1] is given by the percentage of the shares of company *j* owned by each shareholder *i*, where the vector of company *j*’s stakes is denoted by *x*_*j*_ = (*x*_1*j*_, …, *x*_*nj*_). We say *i* has *ownership* of company *j* if *i* owns non-zero equity stakes of *j*, or if *x*_*ij*_ > 0.

A shareholder denoted by *k* ∈ *N* is a third-party to the *ij* relations. Suppose that *i* has no direct ownership of *j* (i.e., *x*_*ij*_ = 0), but *k* has ownership of *j* and so does *i* of *k* (i.e., *x*_*ik*_ > 0, *x*_*kj*_ > 0). Then, by transitivity, *i* indirectly owns *j* through *k*’s ownership of *j* (i.e., ${\bar{x}}_{ij}>0$). It is straightforward to quantifies how a shareholder’s investment in company *j* extends through the ownership network to reach a distant company *k*.

The distance between a shareholder *i* and a target company *j* is defined as the number of nodes along the shortest path from *i* to *j* and is denoted by *d*(*ij*). The direct ownership in the *ij* relations implies *d*(*ij*) = 1 and the indirect ownership implies *d*(*ij*) > 1.

#### 2.5.2 Model of corporate control

The size of equity ownership does not immediately translate into the capacity of managerial control. We derive a *control linkage* *y*_*ij*_ ∈ {0, 1}, from *x*_*ij*_ and target company *j*’s *quota* (i.e., the minimum number of votes required to make a decision) denoted by *q*_*J*_ ∈ (0, 1] such that *y*_*ij*_ = 1 if *x*_*ij*_ ≥ *q*_*j*_; and *y*_*ij*_ = 0 otherwise.

**Fig 1:**
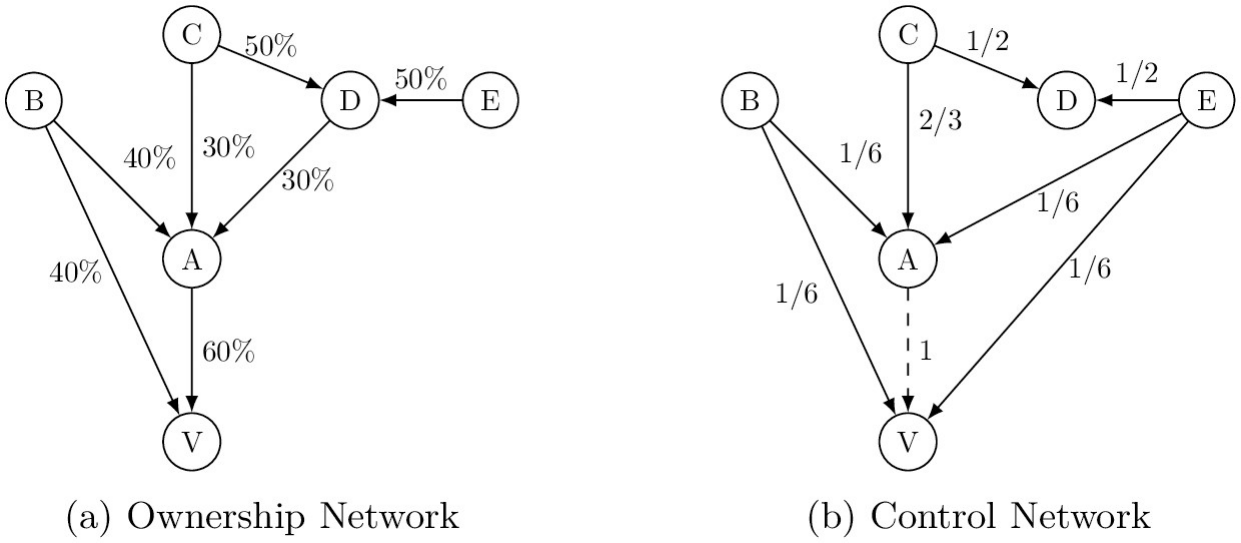
Indirect ownership & control in the shareholding network.

Assuming the simple majority rule, *q*_*j*_ = 1/2, for any *j*’s decision-making, with its 50% share of *D* in Fig 1, the probability that *C* seizes the full control of *D*’s 30% share in *A* is 1/2. This means that with probability 1/2, *C* can consolidate its direct ownership of the 30% share in *A* with the indirect ownership of *D*’s 30% share (i.e., ${\bar{x}}_{CA}=x_{CA}+x_{DA}=30\%+30\%=60\%$), achieving the majority ${\bar{x}}_{CA}>q_{A}$ and hence obtaining the power to fully control *A*’s decision-making (i.e., ${\bar{y}}_{CA}=1$) with probability 1/2. Similarly, since *A*’s direct ownership of *V* exceeds *q*_*V*_ = 1/2, it controls *V*, or ${\bar{y}}_{AV}=1$ with probability 1. By transitivity, therefore, *C* obtains the power to indirectly control *V* through its (probabilistic) control of *D* and *A* as shown in Panel (b) of Fig 1.

Generalizing the power index in this example, Mizuno et al. (2020) proposed a measure for ultimate owners *i*’s power to control managerial decisions of firm *j* based on *i*’s direct or indirect shareholding in the ownership network, called the *Network Power Index* (NPI), [4]. This measure for the power of corporate control can be interpreted as an extension of Shapley and Shubik’s (1954) voting power index in weighted voting games to the network context [10]. The NPI is defined for every *ultimate owner* *i* with respect to every other *j* as the sum of the (*ex ante*) probability that *i* forms a coalition among other shareholders of *j* (either by consolidation or transitivity) which collects enough voting rights attached to their “combined” ownership ${\bar{x}}_{ij}$ to meet the quota for *j*’s decision-making *q*_*j*_. Formally, individual NPI in the *ij* relations is given as

$$\begin{array}{c} p_{ij}=\sum \mathrm{Pr}({\bar{y}}_{ij}=1|{\left\{{\bar{y}}_{i,k}\right\}}_{i,k})\mathrm{Pr}\left({\left\{{\bar{y}}_{i,k}\right\}}_{i,k}\right), \end{array}$$ (8)

where ${\bar{y}}_{ik}$ denotes the path(s) of control linkages from *i* to *k* in the upstream of an ownership network leading to *j*.

In the example above, ultimate owner *C* can establish the full control over *V* in two scenarios. One is through controlling *D* with probability 1/2, where the *C*’s coalition with *D* has the voting power of 60% to control *A*. Another is when it fails to control *D*, in which case *C* has 1/3 chance of controlling *A*. Since whoever controls *A* also controls *V* due to *x*_*AV*_ = 60% > *q*_*V*_ = 1/2, *C*’s individual NPI with respect to *V* is $p_{CV}=\frac{1}{2}\cdot 1+\frac{1}{2}\cdot \frac{1}{3}=2/3$. Recalling that *C* also controls itself, the sum of the probabilities that *C* controls shareholders and companies in the ownership network gives *C* aggregate NPI value, which is given by $p_{C}=\Sigma_{j}p_{Cj}=p_{CC}+p_{CD}+p_{CA}+p_{CV}=1+\frac{1}{2}+\frac{2}{3}+\frac{2}{3}=\frac{17}{6}$.

The NPI explicitly takes into account both the possibility that centralized (proxy) voting behavior among dispersed shareholders converts fragmented ownership into corporate control and the sequence of consolidated controls channeled through an ownership network. For the actual calculation of NPI and the interpretation of the results, three issues are worth mentioning here. First, Eq (8) indicates that the structure of control over company *j* is conditional on the structure of control over intermediaries *k* in the upstream of the ownership network. Second, NPI quantifies shareholders’ *potential* to control corporate decision-making and it does not necessarily imply that they will actually exercise their power in practice. For other important qualifications of NPI, see [4], Third, the NPI assumes that *i*’s structural power of control over *j* does not decrease in the distance between *i* and *j*, i.e., *d*(*ij*).

#### 2.5.3 Algorithm

While the definition of NPI is straightforward, its calculation is not. The calculation of the NPI value for each shareholder *i* over every other company *j* must take into account every possible path of control linkages through which *i*’s power of influence might extend to *j* through every *k*. Moreover, because cyclic ownership is pervasive often due to the practice of cross-shareholdings and because our model of NPI presumes the probabilistic element in the structure of corporate control, we use a simulation-based approximation algorithm of NPI called *label propagation* described by [4]. The calculation errors are inevitable but the reliability of our algorithm is ensured via simulations. See [11] for the simulation results of NPI.

## 3 Indirect investing in non-ESG firms: The case for shareholders

We first examine how owning shares of one company may indirectly invest in non-ESG firms even if the shareholders do not intend to do so, where the shareholders’ equity shares in one company may be used to invest in non-ESG firms. To examine how shareholder’s investment indirectly reach the non-ESG firms, we will first focus on the ultimate owners in this section. The ultimate owners are the shareholders who are not owned by any other shareholders. In the stylized network shown above in Fig 1, shareholders *B* and *C* are the ultimate owners. Hence, if non-ESG firms are located at the very end in the downstream of ownership network, say *V* in Fig 1, then the ultimate owners are *in theory* located in the furthermost of upstream in the network. We will then examine the intermediaries between the ultimate owners and non-ESG firms.

### 3.1 Ultimate owners and connectivity to non-ESG firms

The first result we report is the connectivity of the influential ultimate owners to Non-ESG firms. Recall that the ownership network (*N*, *x*_*ij*_) is a direct network, where capital can flow downstream, either directly or indirectly, from ultimate owners *i* to non-ESG firms *j* through a sequence of ownership linkages *x*_*ij*_ > 0. We say ultimate owner *i* is directly connected to non-ESG firm *j* if *x*_*ij*_, and indirectly connected ${\bar{x}}_{ij}>0$ through intermediary *k* if *x*_*ik*_ > 0 and *x*_*kj*_ > 0 so that there exists a path *i* → *k* → *j*. However, we say *i* is *not* connected to *j*, for example, when *i* → *k* ← *j*, where *x*_*ik*_ > 0 and *x*_*jk*_ > 0 but *x*_*kj*_ ≯ 0. This is because, in this last case, there exists no path through which capital flows downstream from *i* to *j*; rather it stops at *k*.

Since our global ownership dataset contains millions of publicly non-listed entities including small companies and individual shareholders and the vast majority of them do not have network connectivity in meaningful ways, we focus on the influential ultimate owners that may have capacity to impact global financial economy. Specifically, we adapt the cutoff of top 10,000 companies in terms of the NPI values weighted by the target company’ operating revenue in billion U.S. dollars.

Of those 10,000 influential ultimate owners, 5,503 shareholders (i.e., about 55%) are connected to at least one non-green firm, while 2,163 shareholders (i.e., about 22%) are eventually connected to arms manufacturers in the ownership network. However, not all non-ESG firms are under (indirect) influence of those large ultimate owners; the number is actually small. More than 50% of 46,539 non-green firms (i.e., 23,971) and 19% of 1,741 arms manufacturers are financially connected to the influential ultimate owners.

While these proportions appear relatively small, very large corporations are more tightly connected to non-ESG firms. Table 1 shows a breakdown of the ultimate owners’ connectivity to non-ESG firms according to their market dominance. The first row shows that *every single* ultimate owner whose NPI values are among top 10 is ultimately connected to *both* the non-green firms *and* the arms manufacturers. The proportion of the ultimate owners connected to non-ESG firms gets smaller as the ultimate owners become less influential in the ownership network. For non-green firms, this proportion reduces from 100% to 52% as the weighted NPI values reduce from among top 10th to top 10,000th. Similarly, the number of the ultimate owners connected to the arms producers more quickly decreases as they became less influential, namely while 89% of the ultimate owners with the 11th through 100th highest NPI values are connected to arms manufacturers, only 19% of those ranked the 1001st through 10,000 are connected to arms manufacturers. Table 2 lists the ultimate owners whose NPI values are among the top 10 in 2016, who were connected to the firms that are identified as non-ESG in 2020.

**Table 1:** Influential ultimate owners connected to non-ESG firms.

Rank of NPI	Non-Green Firms	Arms Manufacturers
Top 1–10	1.00	1.00
11–100	1.00	0.76
101–1,000	0.85	0.44
1,001–10,000	0.52	0.19

*Note*: Entries are the proportion of the ultimate owners

**Table 2:** Shareholders with top-10 NPI in 2016.

Rank	Ultimate Owner	NPI ($v_{j}{\hat{p}}_{i}$)
1	Government of China	7,392.64
2	Government of Norway	2,617.73
3	Capital Group Co. Inc.	2,432.02
4	Wellington Management Group LLP	2,050.74
5	Government of South Africa	1,787.87
6	Vanguard Index Funds	1,479.67
7	Sun Life Financial Inc.	1,166.81
8	Government of the Russian Federation	1,049.08
9	Johnson Family	1,043.85
10	Sumitomo Mitsui Trust Holdings, Inc	989.42

*Note*: Entries are the NPI values weighted by operating revenue in U.S. billion dollars

#### 3.1.1 Shortest path length from ultimate owners to non-ESG firms

**Fig 2:**
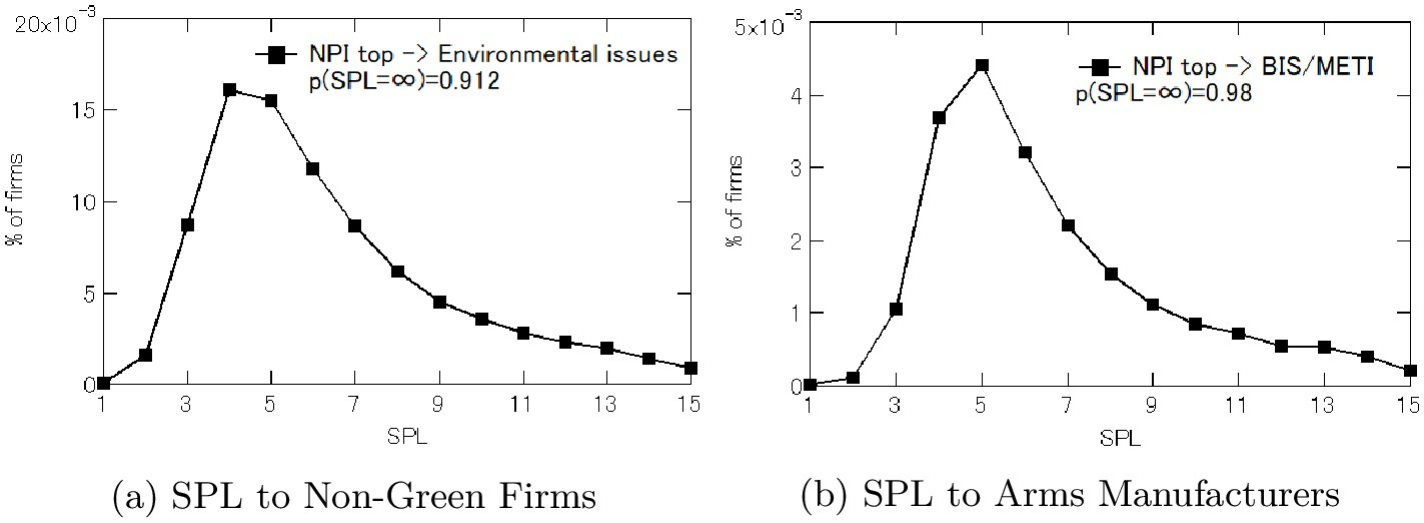
Degrees of separation between top 10K NPI ultimate owners and non-ESG firms.

Now the question is how far away those connected ultimate owners are from those non-ESG firms. We calculate the degree of separation between the ultimate owners and the non-ESG firms by the length of the geodesic path (or the shortest path), that is defined as the minimum number of edges between those two entities in the global ownership network. Fig 2 shows the result for the ultimate owners whose weighted NPI values are, again, among top 10,000.

The first important result here is that none of those companies were directly connected to non-ESG firms, meaning that the influential ultimate owners did not own shares of the firms that are reported by Dow Jone’s Adverse Media in 2020 as having environmental issues or the firms that are identified by the 2020 sanction list maintained by the American or Japanese government as contributing to the production of weapons of mass destruction. In terms of direct stock ownership, therefore, they appear to comply with the ESG standard.

Good news about ESG compliance, however, no longer holds if we look beyond the single degree of separation. Fig 2 shows those influential ultimate owners were indeed indirectly linked to non-ESG firms. Panel (a) indicates that *p*(SPL = ∞) = 0.912, suggesting that the connectivity holds on 8.8% of all the possible correspondences between the influential ultimate owners *i* and the non-green firms *j*. Hence, there exist a path connecting ultimate owner *i* to a non-ESG firm *j* (i.e., ${\bar{x}}_{st}>0$) for the total of *n*(*s*) × *n*(*t*) × 0.088 correspondences.

When the ultimate owners are linked to non-green firms, the most frequent degree of separation is between 3 and 4. Similarly, with respect to arms manufacturers, Panel (b) indicates *p*(SPL = ∞) = 0.98, meaning that the connectivity is identified in 2.0% of all the correspondences that could exist between the influential ultimate owners *i* and the arms manufacturers on the BIS/METI lists. In other words, even though none of the top 10,000 influential ultimate owners appears to be financially connected directly to the problematic arms manufacturers back in 2016, 2,163 of those are indirectly linked to the arms producers on the sanction lists with the shortest path length of more than one. Looking through the arms manufacturers perspectives, 343 of the 1,741 firms receive investments that originate at least in theory from those influential ultimate owners. Interestingly, the most common degree of separation between the influential ultimate owners and the arms manufacturers more or less coincides with that of SPL for non-green firms, which is between 3 and 4.

### 3.2 Intermediate shareholders and connectivity to non-ESG firms

The results so far on indirect investments in non-ESG firms shown in Table 1 and Fig 2 focus on the *ij* relations, i.e., the ultimate owners’ indirect influence on target companies. This section now turns to (indirect) investment in the *kj* relationship, i.e., investments by the intermediate shareholders in the non-ESG firms in the ownership network. Exploring the intermediate shareholder’s connectivity is important because a natural question that arises from the result in the previous section is who is responsible for “bridging” the ultimate owners to non-ESG firms. It is not entirely clear whether those ultimate owners (including large financial institutions) who are 4 to 5 degrees of separation have full and complete information about, let alone full control over, where the capital injected by their stock investment flows into.

To address this question, we calculate the *betweenness centrality* [12] for all the shareholders (including the ultimate owners and target companies alike) between the non-ESG firms and the ultimate owners whose NPI values (weighted by the operating revenue of the target companies) are ranked among top 1,000. This analysis would show who is “bridging” the most between the influential ultimate owners and non-ESG firms. The results are summarized in Tables 3 and 4, each of which lists the shareholders with the highest scores of the betweenness centrality respectively for the non-green companies and the arms manufacturers.

**Table 3:** Top 20 “bridges” to non-green firm.

Rank	Between Centrality	Country	Company Name
1	1,572,071	United States	BlackRock Inc.
2	736,835	Great Britain	Old Mutual Plc
3	680,991	Great Britain	Prudential Plc
4	604,795	Germany	Deutsche Bank AG
5	501,839	United States	State Street Corp.
6	478,539	Great Britain	London Stock Exchange Group PLC
7	455,256	Unites States	Bank of America Corp.
8	421,050	South Africa	Standard Bank Group Ltd.
9	419,169	China	Industrial & Commercial Bank of China
10	400,966	United States	Goldman Sachs Group Inc.
11	337,428	United States	Bank of New York Mellon Corp
12	317,048	Japan	JX Holdings, Inc.
13	313,404	Japan	Japan Trustee Services Bank Ltd
14	311,520	Japan	Mitsubishi UFJ Financial Group Inc
15	287,308	China	Guotai Junan Securities Co., Ltd.
16	274,541	United States	Legg Mason Inc.
17	270,407	United States	JPMorgan Chase & Co.
18	254,930	France	BPCE SA
19	254,684	Great Britain	Schroders PLC
20	245,993	Japan	INPEX Corp.
37	177,981	United States	Vanguard Group INC
107	73,865	United States	Fidelity Management and Research LLC

**Table 4:** Top 20 “bridges” to arms manufacturers.

Rank	Between Centrality	Country	Company Name
1	45,410	Russia	Gazprom (OJSC)
2	28,690	United States	BlackRock Inc.
3	17,134	Russia	Gazprom Mezhregiongaz (OOO)
4	10,698	Germany	Deutsche Bank AG
5	8,108	United States	State Street Corp
6	7,871	Russia	Rosneft Oil Company (PJSC)
7	6,807	United States	JPMorgan Chase & Co
8	6,041	Great Britain	Old Mutual Plc
9	5,517	Russia	Surgutneftegas (OAO)
10	4,893	Great Britain	HSBC Holdings Plc
11	4,360	United States	Bank of New York Mellon Corp.
12	3,965	Sweden	Swedbank AB
13	3,780	Great Britain	Prudential Plc
14	3,704	Russia	Stroytransgaz (OJSC)
15	3,484	Japan	Mitsubishi UFJ Financial Group Inc
16	3,551	China	Industrial & Commercial Bank of China
17	3,397	South Africa	Standard Bank Group Ltd.
18	3,198	France	Société Générale SA
19	2,964	Japan	Sumitomo Mitsui Trust Holdings, Inc.
20	2,915	Japan	JX Holdings, Inc.
41	1,140	United States	Vanguard Group Inc.
124	542	United States	Fidelity Management and Research LLC

Table 3 shows that *all* the top 20 “bridges” to non-green firm are financial institutions except for two Japanese oil and gas exploration and production companies JX Holdings (ranked 13th, now ENEOS Holdings) and INPEX Corp (ranked 20th). BlackRock Inc. has by far the highest value of betweenness centrality, which is more than double of the value for Old Mutual Plc, ranked 2nd. Since BlackRock played an important role in injecting investments into the companies with environmental issues back in 2016, it makes sense that BlackRock CEO Larry Fink’s 2020 annual letter had to emphasize that “climate risk is investment risk” [1].

Table 4 lists top 20 shareholders that bridge the ultimate owners to arms manufacturers. Five of them are Russian oil and gas companies (Gazprom ranked 1st and 3rd, Rosneft ranked 6th, Surgutneftegas ranked 9th, and Stroytransgaz ranked 14th), and they are (or previously) state-owned enterprises. Since our study in this article uses the sanction lists maintained by the U.S. and Japanese governments, it makes sense that these Russian companies are identified as having the large betweenness centrality values to the companies responsible for producing the weapons of mass destruction. Ironically, other than these Russian business oligarchs, financial institutions based in the U.S. and Japan as well as their allies, the U.K., Germany, and France, occupy the majority of bridges in the global ownership network that send investments to the arms producers that are deemed against the American and Japanese national interest. It is also interesting that some of the very large American financial institutions are commonly listed both in Tables 3 and 4.

**Fig 3:**
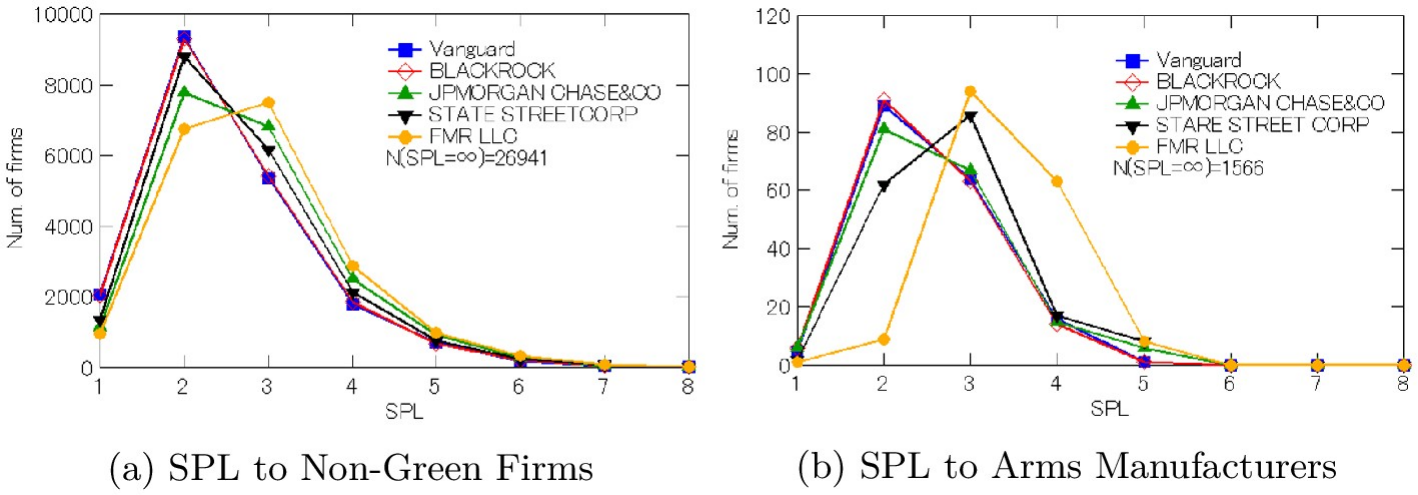
Shortest path length from American financial institutions to non-ESG firms.

We therefore next look more closely at the roles played by BlackRock and other large asset managers based in the U.S. including Vanguard, JPMorgan, State Street, and Fidelity. Fig 3 displays the shortest path length (SPL) from these five asset managers to non-green firms (in Panel (a)) and to arms manufacturers identified by the BIS/METI lists (in Panel (b)). For the companies with environmental issues, 26,941 out of 46,539 companies were not linked to any of these asset managers (i.e., *N*(SPL = ∞) = 26, 941), so that 20,000 of non-green firms (i.e., about 43%) had a link to ownership shares managed by at least one of the five asset managers. Similarly, for arms manufacturers, we find that *N*(SPL = ∞) = 1, 566, which implies that one of the five asset managers managed ownership shares that were connected to 175 of 1,741 arms manufacturers (i.e., 10%).

Fig 3 also shows that these asset managers were much closer to those non-ESG firms than other influential ultimate owners were as suggested by Fig 1. More specifically, Panel (a) of Fig 3 shows that, as of March 2016, all the five financial institutions have the shortest path length of one (1) to at least a thousand non-green firms, meaning that these financial institutions had *direct* ownership of a very large number of companies that had environmental issues. The proximity to non-green firms is even more pronounced if we limit our focus to the “Big Three” asset managers (i.e., BlackRock, State Street, and Vanguard) [6]. The number of the non-green firms that these three fund companies were liked to within one degree of separation jumps up, reaching the order of ten thousands (10K) each. Moreover, the most common SPL with respect to non-green firms was two, so these financial institutions are mostly frequently connected to environmentally problematic companies with just a single degree of separation in the ownership network.

Panel (b) of Fig 3 exhibits the same pattern with respect to arms manufacturers, although the five asset managers rarely had the direct ownership. When they have ownership shares with the link to arms manufacturers, the most common degree of separation for BlackRock, JPMorgan, and Vanguard is one (i.e., SPL = 2), and it is two degrees of separation for State Street and Fidelity. Either way, each of these asset managers had ownership shares that reached more than a hundred arms manufacturers on the sanction lists within single degree of separation.

## 4 Indirect investing in non-ESG firms: The case of equity funds

It is well known that the capital in the ownership network has increasingly been concentrated in the hands of the large financial institutions [3, 6, 13, 14]. However, these financial institutions do not provide the capital on their own. They instead solicit investments into the equity funds that they set up and manage. This leads to the question of which equity funds are linked to the non-ESG firms analyzed in the previous section? To clarify the structure of the ownership and control endowed by the structure of ownership network, we first examine the roles played by the equity funds in the stylized model of ownership network.

### 4.1 Adding equity funds to the ownership network

**Fig 4:**
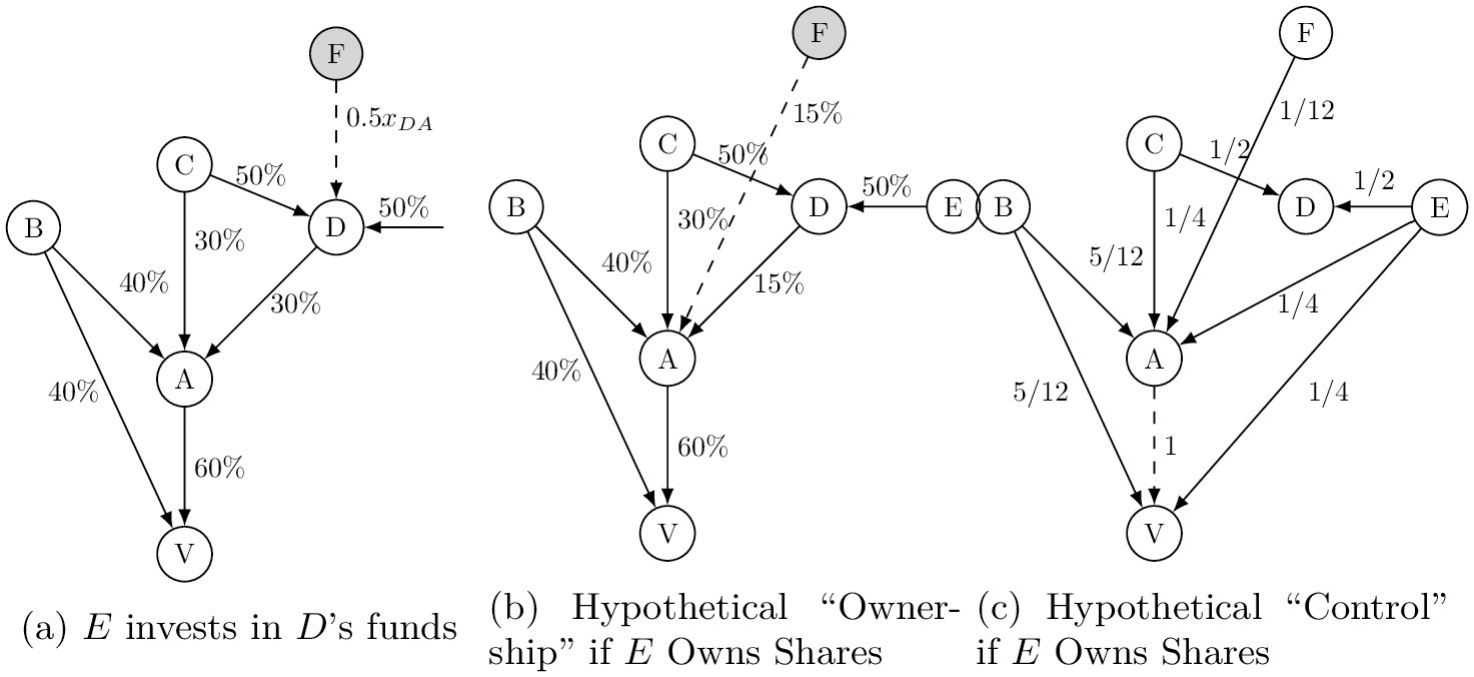
Adding equity funds to the shareholding network: Case 2.

Now consider the extended network shown in Panel (a) of Fig 4, in which shareholder *D* is an investment management company (such as BlackRock or Vanguard Group), who sets up an equity fund (such as an ETF and mutual fund) and is entrusted by investors (denoted by *F*) to manage their investment through the equity funds on their behalf. In doing so, *D* owns, say, 50% of *D*’s share in company *A*. Letting *z*_*ij*_ denote the amount of the equity stake that investor *i* entrusts with fund manager *j* to invest in shares of other company’s stock. In this example, we write *z*_*FD*_ = 0.5*x*_*DA*_. Since investing in shares through equity funds does not involve direct ownership of the shares included in the fund’s portfolio, *F*’s equity stake in *A*’s stock through the equity funds run by *D* does not confer an ownership right in company *A* (unless the investment contract between fund manager *D* and investors *F* stipulates otherwise).

### 4.2 Equity funds and connectivity to non-ESG firms

To explore how the equity funds are linked to non-ESG firms in data, we connect the data on the equity funds provided in *Lipper* database to the ownership network data that we constructed with *Orbis* database. Table 5 summarizes the connectivity of the equity funds to non-ESG firms by types.

**Table 5:** Equity funds connectivity to non-ESG firms.

Type of Funds	Number of Funds	Investment Funds’ Non-Green Firms		Connectivity Arms Producers	
		#	(%)	#	(%)
Managed by BlackRock	2,393	2,157	(93.1)	2,008	(83.9)
Managed by Fidelity	2,208	2,185	(99.0)	1,970	(89.2)
Managed by JPMorgan	1,765	1,646	(93.3)	1,454	(82.4)
Managed by Vanguard	395	391	(99.0)	367	(92.9)
Managed by State Street	263	262	(99.6)	242	(92.0)
Sold in the U.S.	27,996	25,820	(92.2)	22,593	(80.7)
Sold in Japan	1,916	1,793	(93.6)	1,581	(82.5)
Mutual Funds	114,896	108,396	(94.3)	92,810	(80.8)
Insurance Funds	7,946	7,366	(92.7)	6,902	(86.9)
Exchanged-Traded Funds	4,467	4,328	(96.9)	3,732	(85.4)
Pension Funds	1,472	1,404	(95.4)	1,273	(86.5)
Closed-End Funds	483	449	(93.0)	269	(55.7)
Hedge Funds	67	60	(89.6)	46	(68.7)
Advertised as Ethical	8,264	8,175	(98.9)	7,497	(90.7)

The top five rows describe the funds that are managed by BlackRock, Fidelity, JPMorgan, Vanguard, and State Street, giving a real world context to ultimate owner *C* in Panel (a) of Fig 4 through which money is injected into the ownership network by the investors *E*. Almost all of the funds (>99%) managed by Fidelity, Vanguard, and State Street contain in their ownership portfolios the shares that were linked to the firms that were later classified as non-green in 2020. This number is not small (>93%) for two other fund managers: BlackRock and JPMorgan. Similarly, more than 80% of the funds managed by these five American financial institutions back in 2016 contained the ownership shares of the firms that are recognized by the U.S. and Japanese governments as of May 2020 as producing the weapons posing security threats to their nations. Recall that these management companies had a very short path length to the problematic firms, most typically with two to three degrees of separation for arms manufacturers (see Fig 3).

This problem is not peculiar to these large financial institutions; it is ubiquitous for the equity funds sold in the United States and Japan, as shown in the sixth and seventh rows on Table 5. More than 90% of all the funds traded in these two countries were linked to non-green firms and over 80% of them are linked to arms manufacturers.

This financial linkage is slightly attenuated in certain types of equity funds. In the bottom half of Table 5, we classified types of the funds into one of the six categories. Of these, hedge funds were most “green,” while exchange-traded funds (ETFs) and pension funds were least green—that is, the proportion of the hedge funds versus ETFs linked to non-green firms was 89.6% versus 96.9%. With respect to arms manufacturers, closed-end funds and hedge funds are less likely to be linked (with 55.7% and 68.7%), while insurance funds and pension funds are more likely to be connected (with 86.9% and 86.5%) to the firms deemed by the US and Japanese government responsible for producing the weapons of mass destruction.

Perhaps the most interesting is shown at the bottom of Table 5. Almost all the equity funds Refinitive (which used to be a section part of Thomson Reuters section providing Financial & Risk information in 2016) identified as “ethical” were linked to non-green firms (98.9%) and arms manufacturers (90.7%). Our interpretation is that ownership shares included in the portfolio of those “ethical” funds themselves met Refinitive’s requirements for the ethical category; yet, the some of the constitutive shares had indirect linkages (if not directly) to those non-ESG firms.

### 4.3 Equity funds and control over non-ESG firms

Indirect investing in non-ESG firms through equity funds is not only bad news for seeking the sustainability in our society; it might also be good news. Since ownership shares confer the voting rights on the shareholders (and the investors by extension), the investors’ financial linkage to the problematic firms implies that they possibly have a channel to influence managerial decision-making of those firms. As we discussed elsewhere, the NPI measures the power to control the firms in the ownership network [4]. A complication arises in calculating NPI values for the case of equity funds, however, because of the separation of ownership and control, where the equity-fund stakeholders do not hold the ownership of shares (and associated voting rights). In practice, the investment management companies often cast proxy votes on behalf of those equity stakeholders. Note that the kind of the separation of ownership and control that we are concerned with here differs from the one that has been debated in the corporate finance literature [15–18].

To see how the separation of investment and ownership might affect the structure of corporate control, we invoke a hypothetical scenario in which the investors own the shares that are otherwise included in the portfolio of the equity funds. In this hypothetical scenario, the investors’ ownership of the shares now would confer the associated voting rights on them. Consider an extended network in Fig 4 where 50% of asset manager *D*’s share in company *A*’s stock were entrusted by investors *F* (i.e., *z*_*FD*_ = 0.5*x*_*DA*_). This hypothetical scenario posits that *F* owns 50% of *x*_*DA*_ and exercises the associated voting rights on its own. Panel (b) of Fig 4 shows the distribution of ownership share according to this scenario, and Panel (c) shows the distribution of the corresponding hypothetical NPI values.

Against this, we juxtapose the “actual” scenario where we assume the asset managers of the equity funds cast ballots on behalf of the funds’ stakeholders. This is consistent with BlackRock’s practice since 2009, and, according to Fichtner et al., has become a common practice among the Big Three American asset managers [6].

Table 6 summarizes the the distribution of the power of corporate control under the “actual” and “hypothetical” scenarios. It shows that *F* gains the power to control not only the company in which it has equity stakes but also other companies that it does not have any stakes. More specifically, *F* now has individual NPI values of 1/4 with respect to *A* and *V*. Corresponding to this change, fund manager *D*’s share in *A* diminishes by half (i.e., going down from 30% to 15%). But this change in *D*’s shareholding does not affect its power of corporate control since *D* is not an ultimate owner and its shareholding only leverages other shareholders who own *D*. As such, the fact that *D*’s actual shareholding (shown in Panel (a)) is inflated by the contribution of investment money by *F* also affects the power projection capabilities for ultimate owners who do not have immediate relations with *F* but do own *D* who in turn manages the asset on behalf of *F*. Recall that ultimate owners *C* and *E* leverage their ownership in *D* to project their influence over *A* and *V* in the original case (Fig 1). Then, as a consequence of *D*’s loss of its ownership shares in *A* in this hypothetical scenario (Panel (b) of Fig 4), the NPI values for both *C* and *E* decrease from 2/3 to 1/4 with respect to *A* and *V*.

**Table 6:** Individual NPI values for ultimate owners *B* and *C* as well as investor(s) *E*.

	Target Firm	Actual (Fig 1b)	Hypothetical Case (Fig 4c)	
		NPI	NPI	Δ
Investors	D	0	0	0
F	A	0	1/4	+1/4
	V	0	1/4	+1/4
Ultimate Owner	D	0	0	0
B	A	1/6	5/12	+5/18
	V	1/6	5/12	+5/18
Ultimate Owner	D	1/2	1/2	0
C	A	2/3	1/4	−5/12
	V	2/3	1/4	−5/12
Ultimate Owner	D	1/2	1/2	0
E	A	2/3	1/4	−5/12
	V	2/3	1/4	−5/12

This example represents one mechanism through which the power of corporate control is concentrated on a certain group of ultimate owners through the capital-accumulation scheme of equity funds. Table 6 shows that the market influence (measured by NPI) for ultimate owners *C* and *E* is “inflated” by the collecting capital from investors *F* through *D*’s equity funds that are in turn controlled by *C* or *E*.

Moreover, these changes in the power of corporate control propagate through the network since the distribution of NPI across the entire network crucially hinges upon the structure of ownership linkages. In our example (Fig 4), the NPI values for ultimate owner *B* who is not involved in the equity stakes of *F* are also affected, and its NPI increases from 1/6 to 4/9 with respect to *A* and *V*. This is an example where some ultimate owner’s market influence is weakened by the fact that other ultimate owners *C* and *E* inflate their power to control corporations by collecting the capital from investors *F* through *D*’s equity funds.

#### 4.3.1 Approximating the NPI values attributed to the equity funds

Using this theoretical framework, we now attempt to estimate NPI values attributed to the equity funds. This task is difficult since, as we mentioned above, the exact details of investment management contacts between the manager and the client are protected by confidentiality and, as a consequence, there is no way of knowing the identify of those investors (or *F* in Fig 4). We therefore leverage the information on the equity funds provided in *Lipper* database to characterize the NPI values that can be attributed to the clients of those equity funds.

For ease of calculating the hypothetical NPI values for the equity funds, we continue to focus on the five American asset managers: BlackRock, Vanguard Group, State Street, Fidelity, and JPMorgan Chase. These asset managers correspond to shareholder *D* in Fig 4 and the investors whose investments that we try to characterize here corresponds to *F*. To estimate the hypothetical NPI values for investors *F*, we take the share(s) held by the asset managers using the *Orbis* data and subtract the share(s) of the stock included in each fund’s portfolio (i.e., *z*_*FD*_ for investors *F* and the fund managers *D* in our theoretical example) using the *Lipper* data. The hypothetical NPI values are calculated based on the remaining shares held by the fund managers (which corresponds to *x*_*DA*_ − *z*_*FD*_ for *D* in our theoretical model in Fig 4). The amount of the shares that we subtract from the asset managers is then used to calculate the hypothetical NPI values assigned to investors *F* in our theoretical example.

To determine the NPI value for each equity fund managed by these five companies, we divide the shares owned by each of these companies by the equity shares managed by each company. This simple calculation can be justified in this case because the equity funds in the United States are operated by collective investment institutions of a corporate type as opposed to a contract type [19]. Outside of the United States (e.g., the United Kingdom and Japan), investment trusts (operated by contract-type investment fund) are also common along with investment companies.

Since the data do not make available information on the identify of investors *F* in each fund, we instead characterize them in terms of countries where each equity fund is sold. Table 7 lists the countries where those funds are registered for sales. The NPI values are weighted by the operating revenues of the target companies in the U.S. dollars. The NPI values for each country can be interpreted as the structural power that “national” equity stakeholders may collectively have on the ownership network. It is intuitive that the market influence (measured in terms of NPI) is largely concentrated on the funds traded in the United States. This phenomenon simply reflects the fact that we are focusing on the equity funds managed by the American asset managers.

**Table 7:** National influence of the equity funds on non-ESG firms.

Hypothetical NPI	Country	Non-Green Firms			Arms Manufacturers		
		Rank	Hypothetical NPI	%	Rank	Hypothetical NPI	%
5,535,705,203	United States	1	1,420,547,398	25.66	1	7,135,451	0.129
1,449,762,503	Chile	2	373,642,186	25.77	2	2,987,950	0.206
5664,110,77	Peru	3	147,521,073	26.04	3	869,230	0.153
5557,178,23	United Kingdom	5	141,205,303	25.41	7	291,625	0.052
552,252,407	Australia	4	143,868,405	26.05	8	288,896	0.052
242,609,727	Singapore	6	63,799,853	26.30	9	83,066	0.034
142,905,895	Switzerland	7	38,723,713	27.10	12	2,743	0.002
126,231,391	Japan	8	32,485,519	25.73	4	507,608	0.402
110,708,484	France	9	29,855,048	26.97	6	432,057	0.390
101,764,704	Luxembourg	10	27,954,292	27.47	-	-	0
97,739,681	Germany	11	26,179,567	26.78	-	-	0
97,091,010	Austria	12	25,426,802	26.19	10	71,989	0.074
84,662,477	Ireland	13	22,950,708	27.11	5	504,046	0.595
65,297,789	Spain	14	18,460,119	28.27	13	770	0.001

*Note*: If more than one country is denoted for a fund, we split the (weighted) NPI value evenly between the registered countries attached to the fund and distribute them equally among them.

This list also suggests that many of the investors with equity funds through these asset managers reside in the countries that are allies of the United States in the Pacific Rim (e.g., Australia, Japan, Chile) and Western Europe (e.g., the U.K., Germany, and France). It is worth noting that a large number of the equity funds are also registered in Chile and Peru, generating the second and third highest NPI values for Chile and Peru, respectively. This is, however, a reflection of the fact that many equity funds sold by these five American management companies in the United States and Europe are also registered with the regulatory authority of Chile or Peru. As we detailed in Table 14 in S1 Appendix, no fund run by the these asset managers is sold solely in Chile or Peru.

We next examine how much of the influence (in terms of NPI) attributed to the American equity funds is over non-ESG firms. The middle columns in Table 7 list the NPI values with respect to non-green firms, where we see that the NPI values are distributed among the countries almost exactly in proportion to the overall size of power of corporate control (shown in the first column) attributed to the equity funds managed by the five American asset managers. Thus, it is not surprising that corporate control over non-green firms attached to the equity funds is concentrated in the United States. It is also interesting to note that the proportion of the NPI values over non-green firms remains roughly the same across the countries, staying in the [0.25, 0.28] range.

With respect to arms manufacturers, the power of corporate control attached to the equity funds concentrates in the United States; but again this is just a reflection of the concentration of overall NPI in the United States. As for the proportion of the power to control arms manufacturers, it generally remains much lower for all the countries listed here compared to non-green firms, the equity funds sold in Japan, France, and Ireland have higher concentration of influence over arms manufacturers relative to other countries. In Ireland, for example, 0.595% of its NPI value possessed by the “American” equity funds are over arms manufacturers, while for the equity funds sold in Japan the figure is 0.402%. This suggests that individual investors residing in these countries with the equity funds run by the five American asset managers have a higher chance of having influence on the companies responsible for the production of the weapons of mass destruction. On the other hand, the equity funds registered in Germany or Luxembourg do not contain in their portfolio stocks that are linked to arms manufacturers.

**Fig 5:**
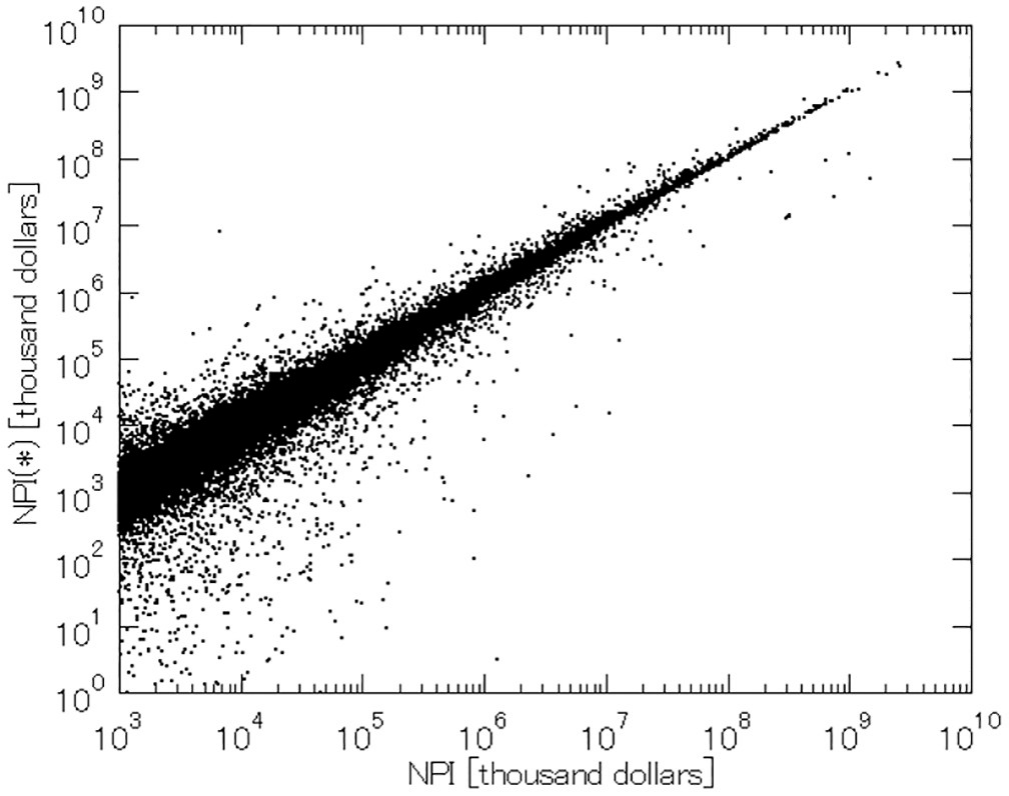
Inflation of NPI values due to equity funds by five American companies.

The last finding that we report is the impact of the market influence attached to the equity funds on the distribution of corporate control in the network. As we have shown in the theoretical example in Table 6, the fact that the investment money concentrates on the asset managers through the equity funds may strengthen the market influence for some ultimate owners, while they may weaken other ultimate owners’ influence. And these impacts on the distribution of the market influence propagate through the ownership network. Fig 5 shows the overall shift in the NPI values if the investors of the equity funds hypothetically own the portfolio shares instead. The horizontal axis represents the original (actual) NPI values and the vertical axis represents the hypothetical NPI values. Both actual and hypothetical NPI values are weighted with the operating revenues (in thousand U.S. dollars) of the target companies. The ultimate owners plotted below the 45-degree line are the ones whose actual market influence is inflated. Thus, there are more ultimate owners whose market influence is enhanced rather than weakened.

**Fig 6:**
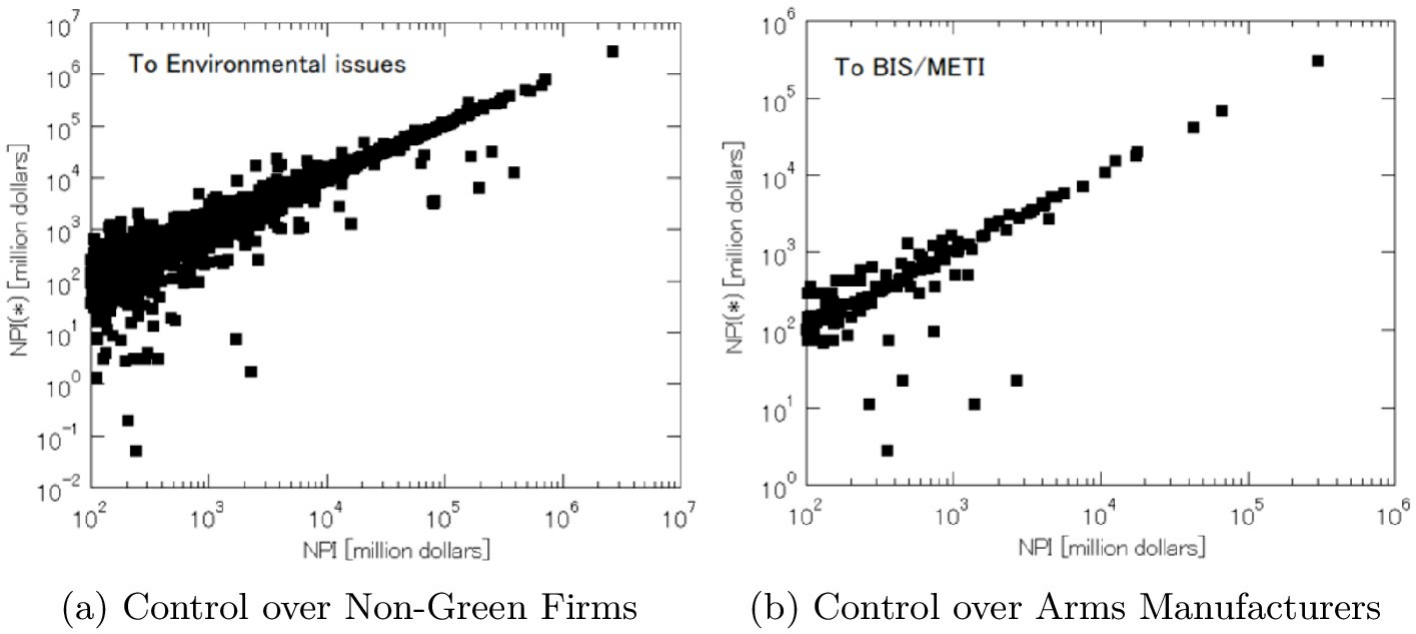
How much equity funds inflate market influence over non-ESG firms.

This pattern also persists if we focus on the market influence over non-ESG firms. In Fig 6, more ultimate owners are plotted below the 45-degree line than above. This implies that the ultimate owners’ power of corporate control is more likely to be inflated than deflated by the concentration of ownership shares around the five American asset managers through their equity funds. And this holds both with respect to non-green firms and arms manufacturers.

To look closer at the ultimate owners who are most empowered with respect to non-ESG firms, we list in Tables 8 and 9 ultimate owners whose NPI values are most inflated as a consequence of investment money being injected into the ownership network through the equity funds run the five American asset managers. Interestingly, six Vanguard group companies are ranked among top 20 for non-green firms and arms manufacturers alike. In Table 8 all the Vanguard group companies’ hypothetical NPI values are somewhere around 3% or 4% of the actual NPI values, meaning that about 96% to 97% of their power of corporate control is generated by the capital collected through the equity funds. Perhaps, these Vanguard group companies represent a peculiar pattern since the most of the equity funds are issued by Vanguard Group Inc who is an intermediate shareholder owned by ultimate owners such as Vanguard Index Funds, Vanguard Bond Index Funds, and Vanguard Star Funds, etc. Thus, in our stylized example (Fig 4), Vanguard Group Inc corresponds to shareholder *C* who is owned by ultimate owners *C* who correspond to these six group companies.

**Table 8:** Equity funds and inflated corporate control over non-green firms.

Rank	Actual NPI	Hypothetical NPI	Δ% in NPI	Country	Company Name
1	83.2E+06	2.5E+06	3.00	United States	Vanguard Fixed Income Securities Funds
2	392.5E+06	13.2E+06	3.38	United States	Vanguard Index Funds
3	81.5E+06	2.9E+06	3.58	United States	Vanguard Specialized Funds
4	197.3E+06	7.1E+06	3.61	United States	Vanguard Bond Index Funds
5	83.1E+06	3.3E+06	4.01	United States	Vanguard Star Funds
6	79.6E+06	3.5E+06	4.37	United States	Vanguard Money Market Reserves
7	165.9E+06	1.3E+06	8.08	Ireland	F.B.D. Trust Company Ltd.
8	16.2E+06	1.8E+06	11.25	Ireland	Farmer Business Developments
9	254.8E+06	29.0E+06	11.40	United States	Johnson Family
10	12.9E+06	2.1E+06	16.62	Canada	Black Creek Investment Management
11	67.0E+06	25.4E+06	37.88	Taiwan	Mr. Tai-Ming Guo
12	12.9E+06	4.9E+06	39.71	Canada	Public Sector Pension Investment Board
13	63.0E+06	27.0E+06	42.89	Norway	Government Pension Fund of Norway
14	13.5E+06	6.0E+06	44.09	United States	Ruane Cunniff & Goldfarb
15	11.1E+06	5.7E+06	51.35	United States	Bahl & Gaynor Inc
16	32.0E+06	16.5E+06	51.57	United States	Vulcan Value Partners LLC
17	528.7E+06	281.5E+06	53.24	United States	Wellington Management Group LLP
18	10.5E+06	6.2E+06	58.63	United States	Cortland Advisers LLC
19	22.0E+06	13.0E+06	58.87	Canada	Manulife Financial Corp
20	18.6E+06	11.1E+06	59.72	France	Caisse d’épargne et de prévoyance

*Note*: Hypothetical NPI assumes that ownership shares included in the equity funds managed by the five American fund managers are owned and exercised by the investors, not by the managers.

**Table 9:** Equity funds and inflated corporate control over arms manufacturers.

Rank	Actual NPI	Hypothetical NPI	Δ% in NPI	Country	Company Name
1	745E+03	0	0	United States	Vanguard Specialized Funds
2	451E+03	0	0	United States	Vanguard Star Funds
3	360E+03	0	0	Norway	Government Pension Fund of Norway
4	164E+03	0	0	United States	Ariel Capital Management Holdings, Inc.
5	160E+03	0	0	United States	Dodge & Cox
6	126E+03	0	0	France	Crédit Agricole S.A.
7	117E+03	0	0	United States	NBSH Acquisition LLC
8	109E+03	0	0	United States	E.R. Colson, C.J. Daley and G.K. Ramirez
9	102E+03	0	0	France	AXA Assurances IARD Mutuelle
10	101E+03	0	0	Great Britain	Mr. Crispin Odey
11	101E+03	0	0	Sweden	Folksam ömsesidig Livförsäkring
12	119E+03	0.77E+03	0.65	United States	State of New York
13	268E+04	4.40E+04	1.64	United States	Vanguard Index Funds
14	515E+03	11.00E+03	2.13	United States	Vanguard Fixed Income Securities Funds
15	140E+04	4.40E+04	3.14	United States	Vanguard Bond Index Funds
16	377E+03	22.00E+03	5.83	United States	Vanguard Money Market Reserves
17	103E+04	7.20E+04	7.01	Ireland	F.B.D. Trust Co. Ltd.
18	101E+03	11.00E+03	10.84	United States	Fisher Investments
19	125E+04	22.2E+04	17.78	United States	Johnson Family
20	249E+03	72.1E+03	28.90	United States	State of California

*Note*: Hypothetical NPI values indicate the NPI values if the five American financial institutions had not managed the stock funds, and instead those equity stakeholders owned the share on their own.

Not all the ultimate owners with large inflation rates are the fund managers, however. The Canadian and Norwegian public sector pension funds (ranked the 12th and 13th respectively) are good examples, where their NPI values could have been 39.7% and 42.9% respectively of the actual values if the investment money had been used to have ownership shares of the stocks included in those equity funds. The same goes for the business families and individuals including the Johnson Family and Mr. Tai-Ming Guo of Taiwan.

Table 9 shows the same pattern with respect to arms producers. The top 11 ultimate owners with very large (actual) influence over the arms manufacturers would have no connection to them had they owned the shares of the stocks included in the equity funds managed by the five American financial institutions (i.e., the hypothetical scenario). That is, this result indicates that these ultimate owners do not have direct ownership in the stocks with any links to arms manufacturers; however, because some shareholders on their ownership paths downstream manage the equity funds that include ownership shares linked to arms manufactures. As a result, these ultimate owners end up in the position in the network with the power to control the ammunition firms.

The ultimate owners with such “accidental” influences include the Norwegian Government Pension Fund as well as the State of New York (ranked in the 12th) and the State of California (ranked in the 20th). Because of the capital concentration on the five American asset managers through their equity funds, the States of California and New York are made much more influential than otherwise over those arms manufacturers the American and Japanese governments considered as posing security threats. This suggests that perhaps there are much more that the residents and the governments of Norway as well as California and New York can do to achieve a more sustainable society than they realize.

## 5 Discussion

This article aims to make two contributions. First, we examine how corporate ownership and equity funds are (involuntarily) responsible for investment in the firms that are deemed problematic with the ESG (Environment, Society and Governance) standards. While the literature has recognized that “ESG is being incorporated into other portfolio products, such as ETFs” in the last several years [20], and the scholarly literature has exclusively addressed the impact of ESG scores on the financial performance of ETFs, corporate profitability, and investment decision-making among others [21, 22]. Our analysis differs from the existing studies in that we study the connectivity of corporate ownership and investment funds to non-ESG firms, rather than the impact of ESG standards.

Second, our analysis connects the equity funds data to the global ownership data. While each of these subjects—the investment funds and the equity shares—separately have received considerable scholarly attentions, to the best of our knowledge, no prior study exists that has explored how the equity funds inject cash into the ownership network. Previous studies have documented that corporate ownership around the world concentrated had become concentrated around financial institutions [3, 6, 13, 14]. One of our objectives has been to explore the source of the power of global financial market. The study that comes closest to ours is the one that examines how passive investment funds intermediate the ownership structure of the automotive corporations [23]. The present study differs in that our ownership network encompasses any industries and in that our equity funds analysis entails both passive and active investment funds.

This study takes a first step to unravel the machinery of ESG investing. We had to make some assumptions to make our study feasible, which we shall relax in our future research. First, it is beyond the scope of this article to explore whether the shareholders and stakeholders actually exert influence utilizing the power of corporate control measured by Network Power Index. There is a tension at least in theoretical terms, between the structurally endowed power and the power that is actually exerted. Second, we focused on the companies with issues in the area of environment and military weapons. However, it is desirable to address other issues areas of Environment, Society, and Governance.

Third, to ease our data-matching task, we limited our focus on the equity funds to those managed by the five American financial institutions including BlackRock, Vanguard and Fidelity among others. We chose these institutions because they are known to have a very large market share [6]. To generalize the present study by evaluating the NPI values associated with the equity shares managed by other asset managers, we would need to devise a new estimation method that take into account not only the corporate-type investment institutions (such as investment companies) but also the contract-type institutions (including investment trusts commonly operating in the U.K. and Japan).

## 6 Conclusion

This article describes the power that shareholders and other equity stakeholders may have to control companies in the global ownership network. To do so, we link up the data on the equity funds to the data on the global ownership network. This is the first study to do so, to our knowledge. We then have investigated how equity investments are connected to non-ESG firms through the ownership network and how that would generate the power for shareholders and equity stakeholders to control companies including non-ESG firms. The analysis shows that large shareholders are very likely to indirectly invest in non-ESG firms most frequently with the several degrees of separation. Many large financial institutions are responsible for bridging the equity investments to those problematic firms. Of those, five American asset managers included in their equity funds the ownership shares that are liked to non-ESG firms with only a few degrees of separation. The capital concentration on these American asset managers through their equity funds also turn out to have significant impact on the power of corporate control for many “distant” shareholders, empowering the Norwegian Pension fund and the States of California and New York with respect to the companies that are considered by the American and Japanese governments as a threat to their national security.

We offer two broad implications based on the analysis presented in this article. First, in the age of the great connectedness in the globalized capital market, there is more that ESG investing can do to cause a change our society in the world. The shareholders, small or large, appear to possess the power to control companies including non-ESG corporations if the power to control’ attached to their equity ownership are orchestrated. Second, although the market for the investment funds in general, and exchanged-trade funds in particular, has expanded in recent years, those funds separate the equity ownership and the rights to control the target corporation(s), which pose some unexplored complications from the perspective of ESG investing. On one hand, this makes large financial institutions including asset managers more powerful than otherwise had they have to use their own asset to own the shares. On the other hand, the investors in those equity funds through those financial institutions could have exerted their influence on the society rather than only obtain the income/capital gains.

## Supporting information

S1 Appendix (PDF)
