# Supplementary material for: Socially responsible investing through the equity funds in the global ownership network
Source: PLoS One. 2021 Aug 12;16(8):e0256160. doi: 10.1371/journal.pone.0256160 (PMC8360556; doi:10.1371/journal.pone.0256160)
Supplement: S1 Appendix — (PDF) [file pone.0256160.s001.pdf]

# A Online Appendix

Upon acceptance, we will remove this appendix from the article to a separate online appendix.

## A.1 Betweenness Centrality between the Influential Shareholders and Non-ESG Firms

In the main text of the article, we show the betweenness centrality for the ultimate owners with top 1,000 NPI values. Here we show another version of the same result for the "influential" shareholders defined in terms of NSR values as opposed to NPI values. The lists in Tables 10 and 11 show top 20 "bridges" between top 1,000 shareholders (defined in terms of NSR) and non-green firms and arms manufacturers respectively. The primary difference stems from the fact that NPI values can only be defined for the ultimate owners and NSR values can be defined for intermediate shareholders as well. Yet, Tables show essentially the same group of entities.

## A.2 Alternative Assumption on Proxy Voting to Calculate Hypothetical NPI Values.

In the main text of the article, we assume investment management companies cast proxy votes on behalf of their equity funds' stakeholders. For calculating the "hypothetical" NPI values, we further assume that if the equity funds investors directly own the shares of the stocks, they would exercise the associated voting rights on their own. Here, we present the alternative "hypothetical" NPI values, with the assumption that it is not the investment management companies but the issuer of the shares what

| Rank | Between Centrality | Country | Company Name |
|---|---|---|---|
| 1 | 54132 | Russia | Gazprom (Public Joint Stock Co.) |
| 2 | 33302 | United States | BlackRock Inc. |
| 3 | 20427 | Russia | Gazprom Mezhregiongaz (Limited Liability Co.) |
| 4 | 13263 | Germany | Deutsche Bank AG |
| 5 | 10168 | United States | State Street Corp |
| 6 | 9384 | Russia | Rosneft Oil Company |
| 7 | 8126 | United States | JPMorgan Chase & Co |
| 8 | 6582 | Russia | OAO Surgutneftegas |
| 9 | 6538 | Great Britain | Old Mutual Plc |
| 10 | 5302 | Great Britain | HSBC Holdings Plc |
| 11 | 5081 | United States | Bank of New York Mellon Corp. |
| 12 | 4532 | Sweden | Swedbank AB |
| 13 | 4416 | Russia | Stroytransgaz (Public Joint Stock Corp) |
| 14 | 3862 | France | Société Générale SA |
| 15 | 3859 | Japan | Mitsubishi UFJ Financial Group Inc |
| 16 | 3825 | Great Britain | Prudential Plc |
| 17 | 3551 | China | Industrial & Commercial Bank of China (ICBC) |
| 18 | 3523 | South Africa | Standard Bank Group Ltd. |
| 19 | 3369 | Great Britain | London Stock Exchange Group PLC |
| 20 | 3098 | Canada | Manulife Financial Corp. |
| 54 | 1242 | United States | Vanguard Group Inc. |
| 145 | 542 | United States | Fidelity MR LLC |

**Table 10.** Top 20 "Bridges" to Arms Manufacturers (NSR)

would cast proxy votes for the investors. Tables 12 and 13 show the list of ultimate owners who would loose corporate control the most *vis-a-vis* non-green firms and arms manufacturers. The ultimate owners with a large gap between "actual" NPI values and "hypothetical" NPI values can be interpreted as the one that are empowered by the practice of proxy voting by the asset managers.

### A.3 Distribution of Countries for the Equity Funds run by the Five U.S. Asset Managers

## B Statement on Data Access

The *Orbis* database is publicly available from Bureau van Dijk (BvD). We have no special access privileges to this database and other researchers will be able to access the database by obtaining a license contract with BvD (https://www.bvdinfo.com/en-gb/) BvD has many office locations around the world including:

Hoogoorddreef 9, 1101 BA Amsterdam-Zuidoost, The Netherlands

Telephone: +31 (0) 20 5400 100

Email: amsterdam@bvdinfo.com

The *Lipper* database is publicly available from Refinitiv, a publisher of business

| Rank | Between Centrality | Country | Company Name |
|---|---|---|---|
| 1 | 1891432 | United States | BlackRock Inc. |
| 2 | 793953 | Great Britain | Old Mutual Plc |
| 3 | 770199 | Germany | Deutsche Bank AG |
| 4 | 767420 | Great Britain | Prudential Plc |
| 5 | 647456 | United States | State Street Corp. |
| 6 | 549009 | Great Britain | London Stock Exchange Group PLC |
| 7 | 536282 | Unites States | Bank of America Corp. |
| 8 | 439837 | Great Britain | Schroders PLC |
| 9 | 436215 | South Africa | Standard Bank Group Ltd. |
| 10 | 433708 | China | Industrial & Commercial Bank of China - ICBC |
| 11 | 423079 | United States | Goldman Sachs Group Inc. |
| 12 | 407047 | United States | Bank of New York Mellon Corp |
| 13 | 336641 | Japan | Mitsubishi UFJ Financial Group Inc |
| 14 | 333375 | France | BPCE SA |
| 15 | 320427 | China | Guotai Junan Securities Co., Ltd. |
| 16 | 301446 | United States | JPMorgan Chase & Co. |
| 17 | 292202 | Canada | Manulife Financial Corp. |
| 18 | 282437 | Germany | Allianz SE |
| 19 | 280402 | United States | Legg Mason Inc. |
| 20 | 256722 | United States | T. Rowe Price Group Inc. |
| 33 | 163620 | United States | Vanguard Group INC |
| 101 | 75594 | United States | Fidelity Management and Research LLC |

**Table 11.** Top 20 “Bridges” to Anti-Environment Firms (NSR)

information (https://www.refinitiv.com/en/). We obtained access to the database by concluding a license contract with Refinitive Japan. The authors are legally bound by the license contract with Refinitiv, the publisher of the database, to not share as open access the firm-level data we analyze in this manuscript. Any parties wishing to access the Lipper database may do so by contacting Refinitiv (https://www.refinitiv.com/en/contact-sale). More detailed information can be obtained at

- Lipper (as service): https://www.refinitiv.com/en/asset-management-solutions/lipper-fund-performance
- Lipper (as data): https://www.refinitiv.com/en/financial-data/fund-data/lipper-fund-data

Finally, the *Adverse Media Entities* database is publicly available from Dow Jones Risk and Compliance (https://professional.dowjones.com/risk/adverse-media-screening/). Again, we have no special access privileges to this database and other researchers will be able to access the data by obtaining a license contract with Dow Jones to obtain access to their information provision service at

| Rank | Original NPI | "Reduced" NPI | Δ% in NPI | Country | Company Name |
|---|---|---|---|---|---|
| 1 | 186E+05 | 0 | 0 | France | Caisse d'épargne |
| 2 | 392E+06 | 12.0E+06 | 3.07 | United States | Vanguard Index Funds |
| 3 | 197E+06 | 6.24E+06 | 3.17 | United States | Vanguard Bond Index Funds |
| 4 | 814E+05 | 30.2E+05 | 3.70 | United States | Vanguard Specialized Funds |
| 5 | 830E+05 | 33.5E+05 | 4.03 | United States | Vanguard Star Funds |
| 6 | 832E+05 | 34.7E+05 | 4.16 | United States | Vanguard Fixed Income Securities Funds |
| 7 | 795E+05 | 33.5E+05 | 4.04 | United States | Vanguard Money Market Reserves |
| 8 | 161E+05 | 12.6E+05 | 7.78 | Ireland | Farmer Business Developments |
| 9 | 255E+06 | 32.2E+06 | 12.62 | United States | Johnson Family |
| 10 | 166E+06 | 25.4E+06 | 15.28 | Ireland | F.B.D. Trust Co. Ltd. |
| 11 | 129E+05 | 27.4E+05 | 21.24 | Canada | Black Creek Investment Management Inc. |
| 12 | 630E+05 | 187E+05 | 29.75 | Norway | Government Pension Fund of Norway |
| 13 | 670E+05 | 271E+05 | 40.40 | Taiwan | Mr. Tai-Ming Guo |
| 14 | 135E+05 | 74.0E+05 | 54.81 | United States | Ruane Cunniff & Goldfarb |
| 15 | 252E+05 | 174E+05 | 69.29 | Spain | Mr. Florentino Pérez |

**Table 12.** Ultimate Owners That Inflated Market Influence with Equity Funds vis-a-vis Non-Green Firms (Under an Alternative Assumption of Voting Behavior for "E")

| Rank | Original NPI | "Reduced" NPI | Δ% in NPI | Country | Company Name |
|---|---|---|---|---|---|
| 1 | 51E+04 | 0 | 0 | United States | Vanguard Fixed Income Securities Funds |
| 2 | 37E+04 | 0 | 0 | United States | Vanguard Money Market Reserves |
| 3 | 21E+04 | 0 | 0 | Great Britain | Flavida (Bermuda) Ltd. |
| 4 | 13E+04 | 0 | 0 | Belgium | Ackermans & van Haaren |
| 5 | 12E+04 | 0 | 0 | United States | State of New York |
| 6 | 10E+04 | 0 | 0 | United States | Fisher Investments |
| 7 | 25E+04 | 0.005E+04 | 0.02 | United States | State of California |
| 8 | 36E+04 | 0.27E+04 | 0.76 | Norway | Government Pension Fund of Norway |
| 9 | 140E+04 | 1.10E+04 | 0.79 | United States | Vanguard Bond Index Funds |
| 10 | 267E+04 | 2.20E+04 | 0.82 | United States | Vanguard Index Funds |
| 11 | 27E+04 | 1.10E+04 | 4.12 | United States | Geode Capital Holdings |
| 12 | 45E+04 | 2.20E+04 | 4.88 | United States | Vanguard Star Funds |
| 13 | 75E+04 | 9.40E+04 | 12.6 | United States | Vanguard Specialized Funds |
| 14 | 37E+04 | 7.21E+04 | 19.66 | Russia | Mr. Vagitovich Alekperov |
| 15 | 126E+04 | 50.5E+04 | 40.12 | United States | Dimensional Fund Advisors |

**Table 13.** Ultimate Owners That Inflated Market Influence with Equity Funds vis-a-vis Arms Manufacturers (Under an Alternative Assumption of Voting Behavior for "E")

https://visit.dowjones.com/risk/lp/adverse-media/.

**Table 14.** Countries where the Equity Funds are Registered

| Country | # of Funds | Single Country | CL | PE | GB | AU | SG | CH | JP | FR | LU |
|---|---|---|---|---|---|---|---|---|---|---|---|
| U.S. (US) | 3,128 | 2,818 | 277 | 223 | 0 | 20 | 72 | 0 | 3 | 0 | 0 |
| Chile (CL) | 1,503 | 0 | - | 301 | 950 | 20 | 716 | 131 | 5 | 1,031 | 1,218 |
| Peru (PE) | 354 | 0 | - | - | 131 | 20 | 144 | 131 | 1 | 131 | 131 |
| U.K. (GB) | 2,443 | 251 | - | - | - | 5 | 809 | 1,809 | 9 | 1,794 | 1,842 |
| Australia (AU) | 55 | 20 | - | - | - | - | 18 | 5 | 0 | 5 | 5 |
| Singapore (SG) | 1,128 | 1 | - | - | - | - | - | 963 | 11 | 929 | 1,010 |
| Swiss (CH) | 2,372 | 1 | - | - | - | - | - | - | 9 | 2,011 | 2,279 |
| Japan (JP) | 118 | 104 | - | - | - | - | - | - | - | 9 | 11 |
| France (FR) | 2,157 | 3 | - | - | - | - | - | - | - | - | 2,004 |
| Luxembourg (LU) | 2,408 | 4 | - | - | - | - | - | - | - | - | - |
